# Prediction of the Strain Response of Poly-AlN/(100)Si Surface Acoustic Wave Resonator and Experimental Analysis

**DOI:** 10.3390/s16050603

**Published:** 2016-04-27

**Authors:** Shuo Chen, Zheng You

**Affiliations:** 1 Department of Precision Instrument, Tsinghua University, Beijing 100084, China; chenshuo_tsinghua@126.com; 2 State Key Laboratory of Precision Measurement Technology and Instruments, Tsinghua University, Beijing 100084, China

**Keywords:** surface acoustic wave resonator, aluminum nitride, strain coefficient factor

## Abstract

The strain sensitivity of the Aluminum Nitride (AlN)/Silicon (Si) surface acoustic wave resonator (SAWR) is predicted based on a modeling method introduced in this work, and further compared with experimental results. The strain influence on both the period of the inter-digital transducer (IDT) and the sound velocity is taken into consideration when modeling the strain response. From the modeling results, AlN and Si have opposite responses to strain; hence, for the AlN/Si-based SAWR, both a positive and a negative strain coefficient factor can be achieved by changing the thickness of the AlN layer, which is confirmed by strain response testing based on a silicon cantilever structure with two AlN configurations (1 μm and 3 μm in thickness, respectively).

## 1. Introduction

Surface acoustic wave resonators (SAWR), which are resonators based on the surface acoustic wave (SAW) generated in piezoelectric materials, have been widely used as key parts of filters in telecommunication. Nowadays, they are also finding roles in the sensing industry, such as in stress/strain sensing, temperature sensing, and gas sensing [1,2,3,4,5]. For the bulk single-crystalline materials, different cuttings of the single-crystalline piezoelectric material or different propagation directions of the SAW may lead to different sensing properties. A good example is the quartz SAWR, where the ST-cut quartz is a good candidate of strain/stress sensing with zero temperature sensitivity, while the YX-cut quartz shows a good sensitivity to temperature.

The most conventional piezoelectric materials for making SAWR are bulk materials such as LiNbO_3_, LiTaO_3_, and quartz, the sensitivity and propagating properties of which have been widely explored. One problem facing these materials is that they are not compatible with the silicon micro-fabrication process, which restricts their on-chip integration with mature Si-based micro-structures. Besides, their working temperatures are also limited, since they all have a phase transaction to a non-piezoelectric material at elevated temperatures [6,7].

Therefore, film piezoelectric materials such as aluminum nitride (AlN) are promising since they can be grown on various substrates such as Si, sapphire and diamond, and they are compatible with the micro-fabrication process to make different types of resonators [8,9]. AlN is also a candidate piezoelectric material for harsh environment applications, with a high melting point, high thermal conductivity and a non-ferroelectric characteristic [10,11].

**Figure 1:**
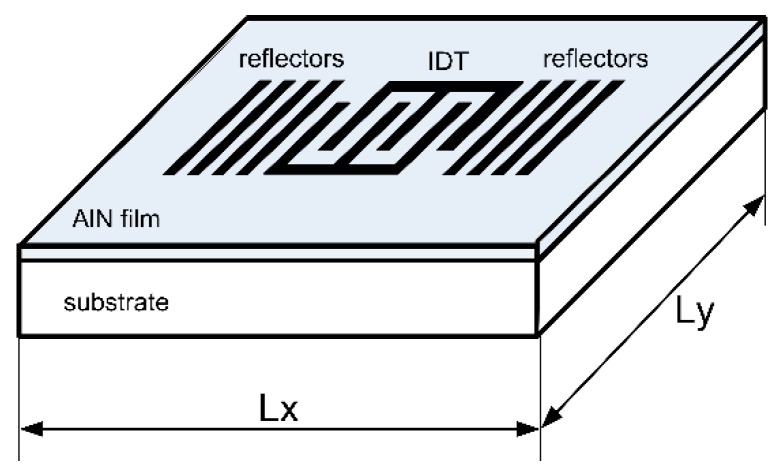
Typical structure of AlN film–based SAWR.

The typical structure of an AlN film-based SAWR is shown in Figure 1 from the top as well as the schematic sides, with dimensional parameters of Lx and Ly as defined for the basal plane. The highly c-axis-oriented polycrystalline AlN film serves as the piezoelectric film and can be deposited on the substrate by sputtering [12]. Another metal layer is patterned into the inter-digital transducer (IDT) and reflectors. The resonant frequency (f_r_) of the SAWR can be expressed as Equation (1), where v_SAW_ is the velocity of the SAW propagating along the IDT, and λ is the period of the IDT.

(1) $$f_{r}=v_{\text{SAW}}/\lambda$$

Most of the reports on the AlN film–based SAW are about the growing of the AlN film and the performance of the resonators themselves, and only several pioneering works have reported the SAWR’s stress/strain response [1,13,14]. Some literature has contributed the sensitivity with strain to the deformation of the substrate and taken the SAW velocity as a constant, which leads to a negative strain coefficient factor (SCF) with resonant frequency, while other researchers have taken the changes of the SAW velocity into consideration by using the pressure-dependent elastic constants, which could be only tested experimentally. The lack of methods for predicting the strain/stress response of AlN film–based SAWR limits the further design and optimization of the AlN film–based strain and pressure sensors.

The purpose of this work is to find a way of predicting the stress/strain response of the AlN/Si-based SAWR, with a comparison of experimental test results. It will show that both negative and positive SCFs with resonant frequency can be predicted theoretically and observed experimentally.

## 2. Theory and Modeling

### 2.1. Responses of Resonant Frequency to Strain

**Figure 2:**
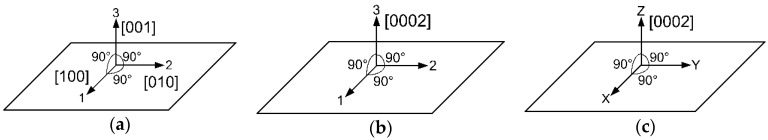
Coordinate systems used in the deriving of SAW velocity ((**a**) cubic; (**b**) single-crystalline hexagonal; (**c**) polycrystalline hexagonal).

Coordinate systems are defined as shown in Figure 2 to better explain the following theory and modeling. Figure 2a,b defines the systems of (100) cubic single-crystalline material (Si in this work) and (0002) hexagonal single-crystalline material, respectively. Figure 2c defines the system of polycrystalline hexagonal material (poly-AlN in this work). The SAW propagates along the 1(or X)-axis in all coordinate systems as defined in Figure 2.

As can be seen from Equation (1), the resonant frequency of SAWR is determined by the period of IDT and the SAW velocity, with a strain field ε, and it can be rewritten as:

(2) $$f_{r,\varepsilon }=v_{\text{SAW},\varepsilon }/\lambda_{\varepsilon }$$

Hence, the SCF of resonant frequency can be expressed as:

(3) $$\text{SCF}=\frac{\Delta f_{r}}{f_{r}\Delta \varepsilon }=\frac{1}{\Delta \varepsilon }\frac{f_{r,\varepsilon }-f_{r,0}}{f_{r,0}}=\frac{1}{\Delta \varepsilon }\left(\frac{v_{\text{SAW},\varepsilon }-v_{\text{SAW},0}}{v_{\text{SAW},0}}-\frac{\lambda_{\varepsilon }-\lambda_{0}}{\lambda_{0}}\right)$$

where λ_ε_ is determined by the designed layout parameters and the strain along the propagating direction of the SAW (ε_SAW_), and can be given by Equation (4), where λ_0_ is the designed period of IDT:

(4) $$\lambda_{\varepsilon }=\lambda_{0}(1+\varepsilon_{\text{SAW}})$$

The derivation of v_SAW,ε_ is more complicated, since the expression of v_SAW_ fits into an implicit relation with the density and elastic constants of the propagating material.

For SAW propagating on in cubic crystals and single-crystalline hexagonal crystals in one direction, the SAW velocities fit Equations (5) and (6), respectively [15,16]:

(5) $$c_{33}c_{55}{\left(\rho_{c}v_{\text{SAW}}\right)}^{2}\left(c_{11}-\rho_{c}v_{\text{SAW}}\right)=\left(c_{55}-\rho_{c}v_{\text{SAW}}\right){\left[c_{33}(c_{11}-\rho_{c}v_{\text{SAW}})-c_{13}{}^{2}\right]}^{2}$$

(6) $$c_{33}c_{55}{\left(\rho_{h}v_{\text{SAW}}\right)}^{2}\left(c_{11}-\rho_{h}v_{\text{SAW}}\right)=\left(c_{55}-\rho_{h}v_{\text{SAW}}\right){\left[c_{33}(c_{11}-\rho_{h}v_{\text{SAW}})-c_{13}{}^{2}\right]}^{2}$$

where c_ij_ represents the elastic constants of the material (i, j = 1, 2, 3), and ρ_c_ and ρ_h_ are, respectively, the densities of the cubic and hexagonal materials. Thus, the SAW velocity can be expressed as:

(7) $$v_{\text{SAW}}=f(\rho ,c_{\text{ij}})$$

In the aforementioned strain field ε, both the mass density and the elastic constants will be affected, and therefore the SAW velocity can further be expressed as:

(8) $$v_{\text{SAW},\varepsilon }=f(\rho \left(\varepsilon \right),c_{\text{ij}}(\varepsilon ))$$

where ρ(**ε**) changes with the strain field as expressed in Equation (9) in the Euler coordinate system:

(9) $$\rho \left(\varepsilon \right){=\rho }_{0}(1-\varepsilon_{1(X)}-\varepsilon_{2(Y)}-\varepsilon_{3(Z)})$$

The relationship between strain and elastic constants for single-crystalline materials can be expressed by third-order elastic constants, the theory of which has been well studied [17,18], and can be expressed as Equation (10) for simplification:

(10) $$c_{\text{ij}}\left(\varepsilon \right)=g(c_{\text{ij}},c_{\text{ijk}},\varepsilon_{1\left(X\right)},\varepsilon_{2\left(Y\right)},\varepsilon_{3\left(Z\right)})\;i,j,k=1,2,3$$

For the polycrystalline AlN used in this work, the c-axis–oriented hexagonal crystallizations (along the Z coordinate axis as shown in Figure 2c) are consistent, while in the film plane (the X-Y plane as defined in Figure 2) their directions are distributed equally in statistics. Therefore, elastic constant $c_{33}$ which is only related to the c-axis of crystallization is the same as single-crystalline AlN; elastic constants $c_{55}$ and $c_{13}$ can also be expressed the same as the single-crystalline ones, since the third-order elastic constants for calculating these parameters are isotropic in the X-Y plane [19], while elastic constant $c_{11}$ shall be recalculated due to the inconsistent third-order elastic constants in one and two directions, and an effective elastic constant $c_{\text{XX}}$ is defined and used in this work. Hence, for polycrystalline AlN, Equation (6) can be rewritten as:

(11) $$c_{33}c_{55}{\left(\rho_{h}v_{\text{SAW}}\right)}^{2}\left(c_{\text{XX}}-\;\rho_{h}v_{\text{SAW}}\right)=\left(c_{55}-\rho_{h}v_{\text{SAW}}\right){\left[c_{33}(c_{\text{XX}}-\rho_{h}v_{\text{SAW}})-c_{13}{}^{2}\right]}^{2}$$

A simplified statistical method is presented here for an estimation of $c_{\text{XX}}$ under a one-directional stress case.

**Figure 3:**
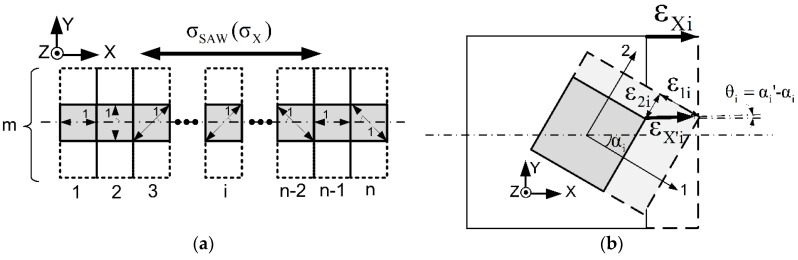
One-directional stress case of a polycrystalline film ((**a**) layout of single-crystalline AlN elements; (**b**) coordinate system of one element).

As shown in Figure 3, each square represents a single-crystalline element of the highly c-axis-oriented hexagonal material projected on the X-Y plane, and has its own single- crystalline coordinate system as defined in Figure 2b. For square element *i*, the angle between 1-axis of the square element and X-axis of the whole film is defined as $\alpha_{i}$. The one-directional stress $\sigma_{X}$ along X-axis can thus be projected to 1-axis and 2-axis as $\sigma_{1X,i}$ and $\sigma_{2X,i}$, and the strain on each axis can be given by:

(12) $$\varepsilon_{1X,i}=\frac{\sigma_{1X,i}}{c_{11}(\varepsilon )}=\frac{\sigma_{X}\cos\alpha_{i}}{c_{11}(\varepsilon )}$$

(13) $$\varepsilon_{2X,i}=\frac{\sigma_{2X,i}}{c_{11}(\varepsilon )}=\frac{\sigma_{X}\sin\alpha_{i}}{c_{22}(\varepsilon )}$$

Therefore, the value of strain vector $\varepsilon_{X\prime ,i}$ and the angle (α′) from it to 1-axis can be given by:

(14) $$\varepsilon_{X\prime ,i}=\sqrt{\varepsilon_{1X,i}{}^{2}+\varepsilon_{2X,i}{}^{2}}$$

(15) $$\alpha_{i}{}^{\prime }=\text{arctan}\left(\frac{\varepsilon_{2X,i}}{\varepsilon_{1X,i}}\right)\;=\text{arctan}\left(\frac{c_{11}(\varepsilon )}{c_{22}(\varepsilon )}\tan\alpha_{i}\right)$$

As can be seen from Equation (15), there will be an angle between the actual direction of strain (X’) and X-axis, namely $\theta_{i}$, which can be omitted, since it is a high-order minimum which comes from the difference of higher-order elastic constants of AlN in 1-axis and 2-axis; hence, the effective strain $\varepsilon_{X,i}$ along X-axis can be expressed as:

(16) $$\varepsilon_{X,i}=\sqrt{\varepsilon_{1X}{}^{2}+\varepsilon_{2X}{}^{2}\;}=\sqrt{{\left(\frac{\sigma_{X}{\text{cos}\alpha }_{i}}{c_{11}(\varepsilon )}\right)}^{2}+\;{\left(\frac{\sigma_{X}{\text{sin}\alpha }_{i}}{c_{22}(\varepsilon )}\right)}^{2}}$$

The total strain $\varepsilon_{X}$ can thus be expressed as the combination of strains derived from all elements along X-axis:

(17) $$\varepsilon_{X}=\frac{1}{L}\underset{n}{\sum }\frac{L}{n}\varepsilon_{X,i}=\frac{1}{n}\underset{n}{\sum }\varepsilon_{X,i}$$

Since the sputtered polycrystalline AlN film is isotropic in the X-Y plane, $\alpha_{i}$ varies equally between 0° and 90° in the statistics. Hence, the effective elastic constant $c_{\text{XX}}$ can be expressed as Equation (18) by combining Equations (16) and (17):

(18) $$c_{\text{XX}}=\frac{\sigma_{X}}{\varepsilon_{X}}=\frac{\sigma_{X}}{\frac{1}{n}\sum_{n}\sqrt{{\left(\frac{\sigma_{X}{\text{cos}\alpha }_{i}}{c_{11}(\varepsilon )}\right)}^{2}+{\left(\frac{\sigma_{X}{\text{sin}\alpha }_{i}}{c_{22}(\varepsilon )}\right)}^{2}}}\overset{\begin{array}{c} n\to \infty ,\\ a\sim N(0{}^{\circ},90{}^{\circ}) \end{array}}{\Rightarrow }\frac{\sqrt{2}c_{11}{(\varepsilon )c}_{22}(\varepsilon )}{\sqrt{c_{11}{\left(\varepsilon \right)}^{2}{+c}_{22}{\left(\varepsilon \right)}^{2}}}$$

where $c_{11}(\varepsilon )$ and $c_{22}(\varepsilon )$ can be calculated as the elastic constants of single-crystalline materials given by Equation (10).

With a given strain ε along the propagating direction of SAW, the period of IDT can be calculated by Equation (4), while the mass density (ρ(ε)) and SAW velocity ($v_{\text{SAW},\varepsilon }$) of both Si and poly-AlN under a given strain field can be calculated with Equations (8)–(10) and (18), respectively. The SCF of the resonant frequency can thus be calculated based on Equation (3). All the material parameters used for modeling and calculation are from previously reported literature and are shown in Table A1 [19,20,21].

**Figure 4:**
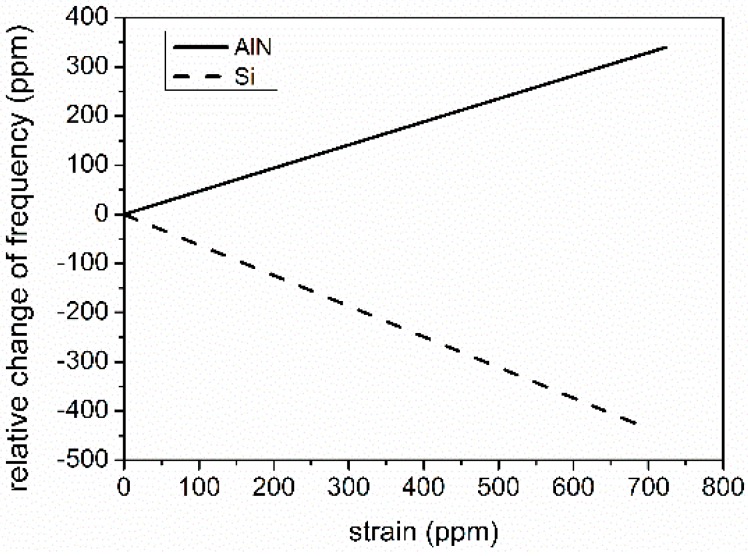
Simulated SCF of resonant frequency for AlN and Si.

As can be seen from Figure 4, AlN and Si are showing opposite SCFs of resonant frequency, indicating different SCFs may be achieved for AlN/Si SAWR with different thicknesses of AlN.

### 2.2. Distribution of Kinetic Energy for AlN/Si SAWR and Its Influence on SCF of SAWR

**Figure 5:**
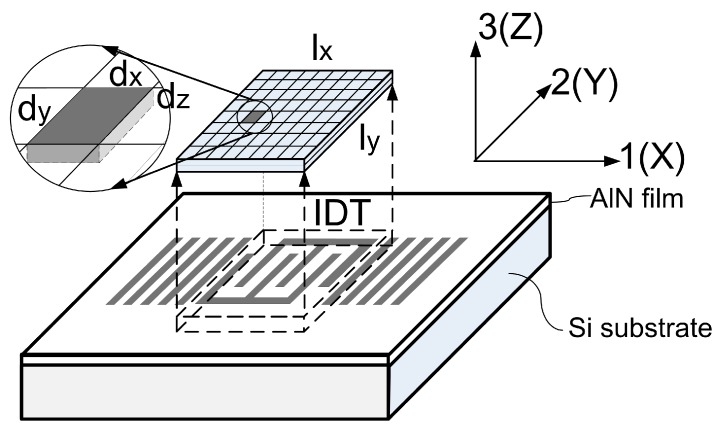
Definitions of micro-units for the calculation of SCF of SAWR.

The electrical energy provided to the IDT is transferred to kinetic energy in the form of mechanical vibrations of both AlN and Si. The distribution of kinetic energy can be calculated based on the definitions of micro-units shown in Figure 5, the coordinate system of which is the same as defined in Section 2.1.

**Figure 6:**
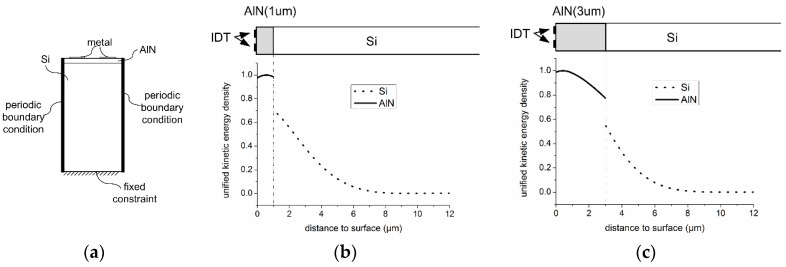
FEM modeling of one periodic SAWR elements and the kinetic energy profile ((**a**) two-dimensional FEM model of SAWR; (**b**) kinetic energy density profile along 3(or Z)-axis of type-A SAWR; (**c**) kinetic energy density profile along 3(or Z)-axis of type-B SAWR).

For an IDT with an aperture (w) much larger than the period (λ), the kinetic energy profile along 3(or Z)-axis can be drawn by the FEM method mentioned in other literature, as shown in Figure 6a, and is not sensitive to 1-2(or X-Y)coordinates [22].

Two stack configurations are modeled by FEM, with type A having a stack of 1 μm AlN/400 μm Si, and type B having a stack of 3 μm AlN/400 μm Si. Both types of SAWR have an IDT period of 12 μm. The simulated profiles of kinetic energy density through the depth of both type A and type B are shown in Figure 6b,c, and a discontinuity of energy densities can be observed, which is due to the different mass densities of AlN and Si. Both stacks are showing an attenuation with an increasing distance to the surface, leading to less kinetic energy, and less influence on the resonant frequency. It can also be observed that most of the kinetic energy is constrained within the surface area of SAWR, and for both type A and type B configurations in this work, less than 0.1% of the kinetic energy stays outside the depth beyond λ (12 μm in this case); hence, the depth of SAWR, namely $D_{\text{SAW}}$, can be set equal to λ.

With the profile of kinetic energy density through depth, and the SCF of resonant frequency for micro-unit dxdydz (${\text{SCF}}_{\text{xyz}})$, which can be calculated with the method mentioned in Section 2.1, the SCF of the SAWR’s resonant frequency can thus be approximately expressed as:

(19) $$\text{SCF}=\frac{\int_{{-D}_{\text{SAW}}}^{0}\iint_{\text{xy}}{\text{SCF}}_{\text{xyz}}k_{\text{xyz}}\text{dxdydz}}{\int_{{-D}_{\text{SAW}}}^{0}\iint_{\text{xy}}k_{\text{xyz}}\text{dxdydz}}$$

where $l_{x}$ and $l_{y}$ are the side lengths of the IDT part in x and y directions, respectively; $k_{\text{xyz}}$ is the unified kinetic energy density of dxdydz, and as mentioned above, it only varies along 3(or Z)-axis, and therefore it can be simplified to $k_{z}$, and Equation (19) can be rewritten as:

(20) $$\text{SCF}=\frac{\int_{{-D}_{\text{SAW}}}^{0}\iint_{\text{xy}}{\text{SCF}}_{\text{xyz}}k_{z}\text{dxdydz}}{\int_{{-D}_{\text{SAW}}}^{0}\iint_{\text{xy}}k_{z}\text{dxdydz}}$$

### 2.3. SCF of Resonant Frequency for AlN/Si SAWR Based on Cantilever Structure

**Figure 7:**
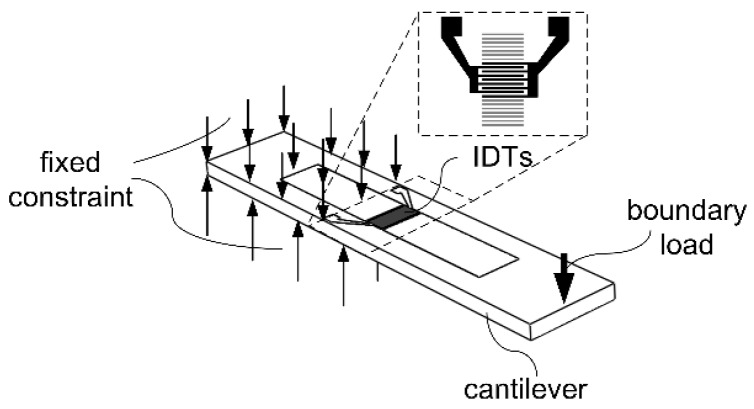
The modeled cantilever structure and its boundary conditions.

Cantilever structures with the same stacks mentioned above (type A and type B) are modeled respectively. For the modeled cantilever structures as shown in Figure 7, fix constraint and load are added at opposite sides of each cantilever, ensuring that the IDT of SAWR positioned at areas with the highest strains.

The designated geometric parameters of the modeled IDT are listed in Table 1, and the strain field of the IDT part ($l_{x}\times l_{y}$) can be derived. Due to the coupled elastic constants, mass density and strain field ε, being coupled with the strain field, finite element modeling (FEM) is utilized for calculating these coupled parameters.

**Figure 8:**
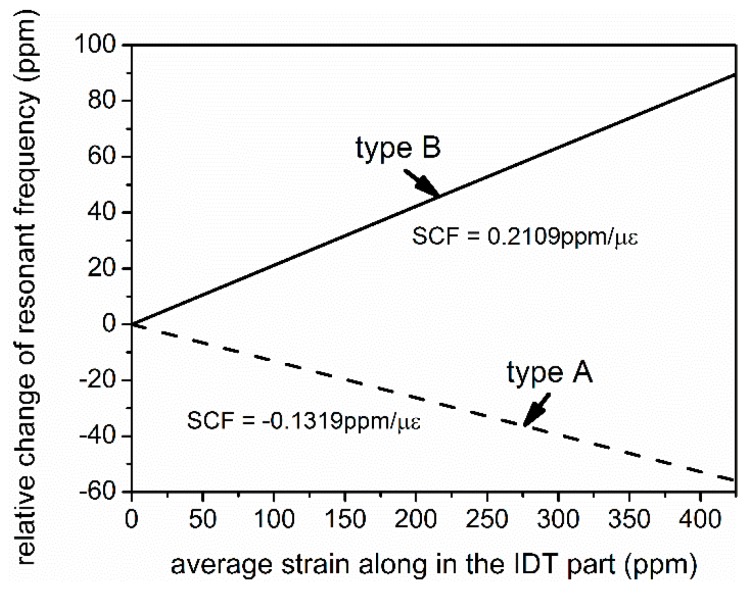
Strain response of both type A and type B SAWR.

The IDT’s SCF of resonant frequency for both type A and type B cantilevers can be calculated based on the method mentioned in Section 2.1 and Section 2.2. As shown in Figure 8, the modeled type A and type B SAWR are showing opposite responses to strains, which is contributed to the opposite SCFs of AlN and Si. The modeling until now has not taken the attenuation or the kinetic energy loss at the AlN/Si interface into consideration, and it can be derived based on experimental results in the following sections.

## 3. Experiment

SAWR samples with two different thicknesses of AlN are prepared, with sample type A having an AlN layer of 1 μm in thickness, and type B having an AlN layer of 3 μm in thickness, which are the same configurations mentioned in previous sections.

**Figure 9:**
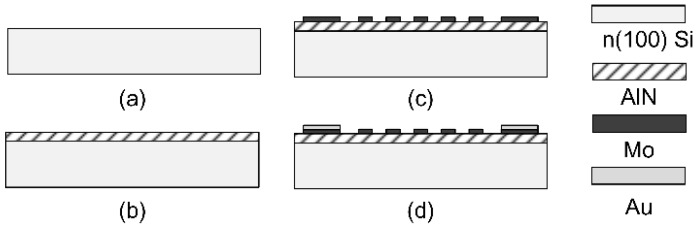
An abbreviated fabricating process of AlN/Si SAWR (not in scale).

Both type A and type B SAWRs are fabricated by the micro-fabricating process, as shown in Figure 9. The 4” *n*-type (100) silicon wafers with a thickness of 400 μm are prepared and cleaned with BHF, acetone, IPA and DI water. A polycrystalline AlN film is deposited on each Si substrate via reactive sputtering, with different thicknesses between type A and B. A molybdenum (Mo) layer of 100 nm in thickness is deposited by sputtering, and patterned into IDT and reflectors by reactive ion etching (RIE). Another layer of 100 nm gold (Au) is evaporated and patterned as electrodes for wire-bonding.

**Figure 10:**
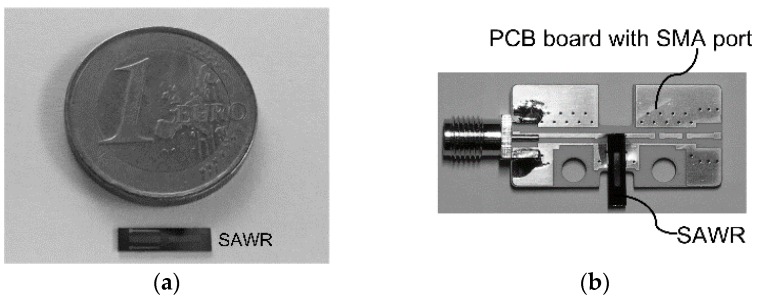
Diced SAWR and the attachment to connection board ((**a**) diced SAWR; (**b**) SAWR with connection board).

After the micro-fabrication, the wafers are diced into SAWR devices with dimensional parameters of L_x_ = 10,400 μm and L_y_ = 2500 μm, as shown in Figure 10a. The temperature sensitivities of the SAWRs are firstly tested with heat applied by a hotplate probe station (Lakeshore TTPX). The testing results are further used to estimate temperature’s influence on the testing results of SCFs.

**Figure 11:**
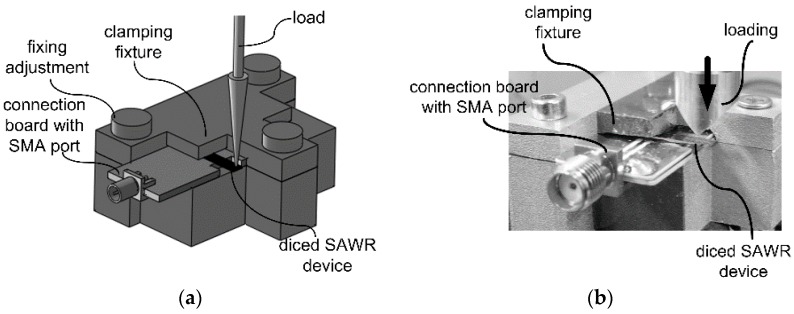
Designed fixtures for the strain response testing ((**a**) designed diagram; (**b**) photo).

The strain/stress response of the SAWRs are tested by the cantilever method, similar to what has been used to determine the gauge factor of semiconductor piezoresistors [23]. The diced SAWR devices are adhered to the connection boards by adhesive agent (Loctite 495) as cantilevers to ensure that IDTs are placed at the clamped edges, where maximum strains are introduced, as shown in Figure 10b. The fixed side of every tested SAWR is further clamped by fixtures designed and fabricated in this work as shown in Figure 11a,b.

**Figure 12:**
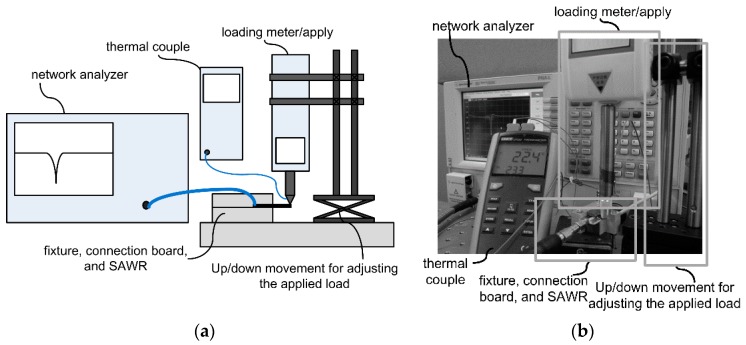
Apparatus for the strain response testing ((**a**) designed diagram; (**b**) photo).

The apparatus used for the strain response testing is shown in Figure 12. Vertical loads reading from 0 N to 1.2 N are applied on the free edge of the fixed cantilevers by loading meter (SHSIWI, SH-30) fixed with a vertical movement platform (BOCIC, VTS101M). FEM method is used to calculate the average strains which are introduced to the IDT of the SAWRs. The responses of the SAWRs in frequency domain are measured by Network analyzer (Agilent Technology, N5230C), with a thermal couple to monitor the temperatures during the testing. The experimental SCFs of resonant frequencies are calculated based on Equation (3) and compared with the predicted results derived through the method introduced in Section 2.

## 4. Results and Analysis

### 4.1. Characterization of AlN/Si SAWR

**Figure 13:**
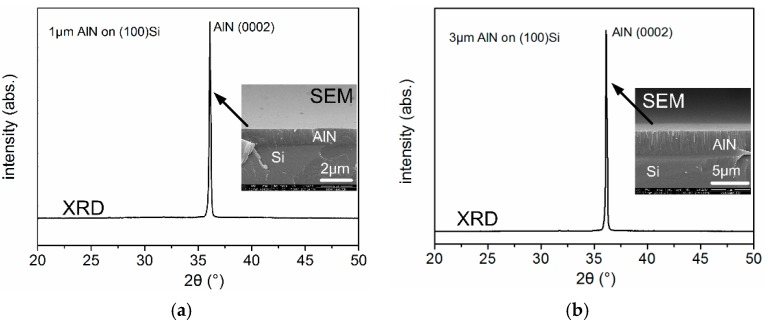
XRD and SEM characterizations of the fabricated type A and type B SAWRs ((**a**) type A; (**b**) type B).

The fabricated SAWRs are characterized by scanning electron microscope (SEM) and X-ray diffraction (XRD) spectrum, as shown in Figure 13, both of which indicate (0002) AlN layers have been grown for type A and type B SAWRS.

**Figure 14:**
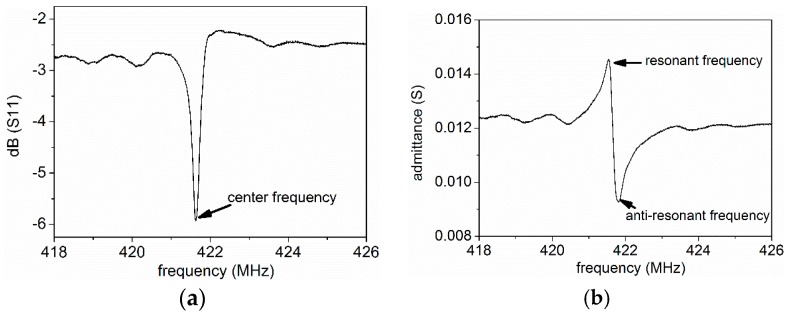
Typical frequency domain response of the fabricated SAWR ((**a**) reflection coefficient; (**b**) admittance).

The typical frequency domain response of the fabricated SAWR is shown in Figure 14. The temperature sensitivity of both type A and type B SAWRs are also tested. SAWRs with type A configuration have an average temperature coefficient factor (TCF) of resonant frequency around 32 ppm/°C, while SAWRs with type B configuration show an average TCF of 29 ppm/°C. These parameters of temperature sensitivity will be used to estimate the temperature’s influences on SCF testing results.

### 4.2. Tested Strain Response of AlN/Si SAWR and Modification of the Model

**Figure 15:**
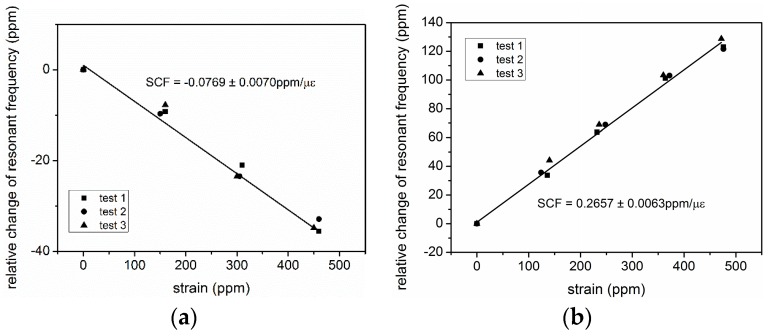
Strain responses of the fabricated SAWRs ((**a**) type A; (**b**) type B).

The strain responses of type A and type B SAWRs are tested based on the cantilever topology shown in Figure 7 and the apparatus shown in Figure 11. The variation of temperature during the testing is less than 0.1 °C; hence, according to the testing results obtained in Section 4.1, the temperature’s impacts on the relative change of resonant frequencies is confined within 3.2 ppm and 2.9 ppm for type A and type B samples, respectively. As can be seen from Figure 15, type A and type B SAWRs are showing opposite responses to strain, which is in accordance with the modeling results in Section 2. First-order approximations have been conducted for the testing results, and the experimental SCFs of type A and type B SAWRs are −0.0769±0.0070 ppm/με and −0.2657±0.0063 ppm/με, repectively. The error parts of the experimental SCFs origin from the uncertainties caused by temperature variation.

**Figure 16:**
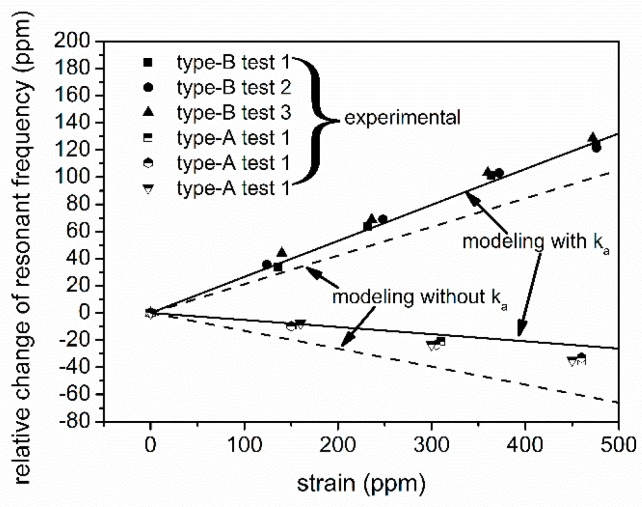
Comparison of the modeling results and experimental results for SCF.

Differences in absolute values exist between the modeling (Figure 8) and the experimental results (Figure 15) due to the attenuation caused by crystalline mismatch and lattice vibration at the interphase of AlN/Si [24,25]. This attenuation may lead to loss of kinetic energy in the Si substrate. A kinetic energy attenuation factor, namely $k_{a}$, is introduced here. Based on the hypothesis that the attenuation happens at the AlN/Si interface, and will affect the unified kinetic energy density of the SAW in Si, Equation (20) can be rewritten as:

(21) $$\text{SCF}=\frac{\int_{{-t}_{\text{AlN}}}^{0}\iint_{\text{xy}}{\text{SCF}}_{\text{xyz}}k_{z}\text{dxdydz}+\int_{{-D}_{\text{SAW}}}^{{-t}_{\text{AlN}}}\iint_{\text{xy}}{\text{SCF}}_{\text{xyz}}k_{a}k_{z}\text{dxdydz}}{\int_{{-t}_{\text{AlN}}}^{0}\iint_{\text{xy}}k_{z}\text{dxdydz}+\int_{{-D}_{\text{SAW}}}^{-t_{\text{AlN}}}\iint_{\text{xy}}k_{a}k_{z}\text{dxdydz}}$$

where $t_{\text{AlN}}$ is the thickness of the AlN layer. As can be seen from Figure 16, by taking an attenuation factor of $k_{a}=0.75$, a better estimation of the strain responses can be achieved, indicating the modeling method introduced here can give a primitive estimation of the strain response of the poly-AlN film–based SAWRs.

## 5. Conclusions

The SCF of resonant frequency for AlN/Si-based SAWRs is predicted based on a modeling method introduced in this work. From the modeling results, AlN and Si have opposite responses to strains, and therefore both a positive and a negative SCF can be achieved by changing the thickness of the AlN layer, which is confirmed experimentally. Further work may focus on widening the scope of this modeling method for AlN layer–based SAWRs on various substrates, and enhancing the design of strain/pressure sensors based on AlN SAWRs.

## Figures and Tables

**Table 1:** Designated geometric parameters of the SAWR.

Parameter	Value (unit)	Parameter	Value (unit)
number of IDT pairs (N_IDT_)	50	w	1200 μm
λ	12 μm	metalizatied ratio	0.5
$l_{x}$	$N_{\text{IDT}}\times \lambda$	$l_{y}$	equals w
$L_{x}$	10,400 μm	$L_{y}$	2500 μm

## Abbreviations

The following abbreviations are used in this manuscript:

SAWR	surface acoustic wave resonator
SAW	surface acoustic wave
IDT	inter-digital transducer
SCF	strain coefficient factor

## Appendix A

The material parameters used for modeling and calculation in this work are listed in Table A1.

**Table A1:** Material parameters used for modeling and calculation.

AlN	Si	Mo
C_11_ = 408.03 (GPa)	C_11_ = 165.63 (GPa)	C_11_ = 440.77 (GPa)
C_12_ = 100.18 (GPa)	C_12_ = 63.87 (GPa)	C_12_ = 172.43 (GPa)
C_13_ = 83.40 (GPa)	C_44_ = 79.55 (GPa)	C_44_ = 121.65 (GPa)
C_33_ = 384.3 (GPa)		
C_44_ = 100.08 (GPa)		
C_66_ = 153.93 (GPa)		
C_111_ = −3072.30 (GPa)	C_111_ = −825 (GPa)	
C_112_ = −514.07 (GPa)	C_112_ = −451 (GPa)	
C_113_ = −75.06 (GPa)	C_123_ = −64 (GPa)	
C_123_ = −155.12 (GPa)	C_144_ = 12 (GPa)	
C_133_ = −614.88 (GPa)	C_166_ = −310 (GPa)	
C_344_ = −576.45 (GPa)	C_456_ = −64 (GPa)	
C_144_ = −150.12 (GPa)		
C_155_ = −100.08 (GPa)		
C_222_ = −2413.0 (GPa)		
C_333_ = −2213.6 (GPa)		
$\rho_{\mathrm{AlN}}$ = 3260 kg/m^3^	$\rho_{\mathrm{Si}}$ = 2329 kg/m^3^	$\rho_{\mathrm{Mo}}$ = 10,200 kg/m^3^
